# Mechanical Response and Microstructure Evolution of TA1 Titanium Under Normal Ultrasonic Vibration Processing

**DOI:** 10.3390/ma18081712

**Published:** 2025-04-09

**Authors:** Yang Liu, Chunju Wang, Haolan Zeng, Xiaoye Liu, Xinhua Song, Zhifang Zhang, Siyuan Liu, Jian Li

**Affiliations:** 1 School of Physical and Electromechanical Engineering, Jishou University, Jishou 416000, China; liuyang@jsu.edu.cn (Y.L.);; 2 School of Mechanical and Electrical Engineering, Robotics and Microsystems Center, Soochow University, Suzhou 215131, China; 3 Department of Aerospace Engineering, Zhangjiajie Institute of Aeronautical Engineering, Zhangjiajie 427000, China

**Keywords:** normal ultrasonic vibration, TA1 titanium sheet, microstructure evolution, mechanical property, softening effect

## Abstract

Ultrasonic vibration (UV) has been employed in various plastic forming processes due to its special effect known as acoustoplasticity. Mostly, UV is applied along the longitudinal direction in experimental investigations. However, very few studies have focused on normal UV-assisted uniaxial tension, which is more similar to the loading state of sheet metal in actual forming processes. Herein, normal UV-assisted tension tests on a TA1 thin sheet are performed to study its mechanical properties and microstructure evolution. The macro-mechanical behavior is demonstrated by stress–strain curves under different ultrasonic amplitudes and strain rates. Fracture morphology and microstructure evolution are characterized by scanning electron microscopy (SEM) and electron backscatter diffraction (EBSD) to reveal the UV softening mechanism at the micro level. The results show that the stress reduction induced by UV reaches 20% when the ultrasonic amplitude is 13.9 μm. Fracture mode changes from ductile fracture to brittle fracture with increasing amplitude. Microstructure examinations show that low-angle grain boundary (LAGB) fraction, kernel average misorientation (KAM), and geometrically necessary dislocation (GND) density in the samples experiencing normal UV-assisted tension are all decreased, leading to a reduction in deformation resistance. The inverse pole figures (IPFs) further reveal that the plastic deformation mechanism of the TA1 thin sheet is diversified with the superposition of normal UV.

## 1. Introduction

Ultrasonic vibration (UV)-assisted forming technology, which merges UV energy with plastic forming processes, has seen widespread use in shaping miniature metal components. Its unique advantages include decreasing resistance to material deformation, changing friction conditions at interfaces, and improving the precision and quality of formed parts. UV-assisted forming technology has gained significant attention in the precision forming of small-scale metal components, such as the successful fabrication of high-quality capillaries [1] and spherical cap arrays [2] using UV-assisted forming. UV has also been superimposed in micro-extrusion [3], micro-punching [4], forging [5], bonding [6], welding [7], and additive manufacturing [8], in which the quality of metal parts was improved. The combination of UV and temperature fields superimposed in the bending process was also reported by Gao et al. [9], and they found that UV notably reduced both forming force and spring-back angle at lower temperatures. Comprehensively, the related studies indicate that UV reduces forming forces and improves surface quality and forming limits in various processes. Specifically, in the plastic forming of miniature components, UV is able to mitigate the challenges and intensified friction due to the size effect, thus enhancing the quality of the formed parts.

Despite extensive studies on UV-assisted forming, the underlying mechanism of the softening effect of metals under UV remains controversial. This complexity arises from the intricate deformation behavior under UV, which encompasses the ultrasonic field, stress–strain field, and size effect. Plastic deformation under UV is influenced not only by process parameters, such as ultrasonic amplitude, ultrasonic frequency, and ultrasonic duration, but also by material properties like crystal structure and dislocation density. Current research suggests that UV impacts plastic deformation behavior primarily through bulk effect and surface effect. Specifically, the bulk effect involves macro-level stress superposition and micro-level acoustic softening. Stress superposition is generally associated with the variation in elastic stress during a single vibration cycle, while acoustic softening refers to the energy absorption of dislocations, which can increase dislocation activity, as reported by Daud et al. [10] and Yao et al. [11]. Wang et al. [12] explored the coupling effect of stress superposition and acoustic softening, establishing a mathematical model of the tensile deformation behavior of copper foil to explain the stress reduction induced by UV. Additionally, further research was conducted on the size effect of UV-assisted micro-forming. A theoretical model of the coupling effect of UV and grain size based on the Hall–Petch relationship was constructed to reveal the microscopic mechanism of the grain size effect under UV from the perspective of energy conservation [13]. The surface effect of UV is related to the interface friction condition between the workpiece and the forming tool. For example, in UV-assisted extrusion [3], the transient separation between the workpiece and the tool due to UV reduces interface contact pressure and friction. Furthermore, the direction of friction changes with the relative motion direction of the workpiece/tool interface. Specifically, when aligned with the extrusion force, friction can facilitate material flow, thereby lowering the forming force. It should be noted that some studies suggest the thermal effect of UV contributes to stress reduction [14], while increasing evidence indicates a negligible temperature rise in metal materials experiencing UV [10,15,16]. Therefore, in the deformation of miniature samples, the thermal effect of UV is typically considered insignificant.

It is worth mentioning that the UV-induced softening effect is typically characterized as an instantaneous phenomenon. However, some experiments have also recorded a residual effect that persists after the UV stops. Both residual softening and residual hardening have been reported in various investigations. Specifically, Yao et al. [11] and Zhou et al. [17] identified residual hardening in pure aluminum compression experiments with ultrasonic assistance. Conversely, Lum et al. [18] observed significant residual softening during the wire bonding of gold, and Wang et al. [16] noted similar residual softening in tensile test of copper foil. Unlike the instantaneous effect of UV, residual effects are often attributed to permanent microstructure changes induced by ultrasonic energy. Thus, residual effects are more easily quantified and characterized through microstructure evolution. For instance, Dutta et al. [19] pointed out that UV can promote dislocation annihilation, thereby reducing dislocation density. Also, both dislocation annihilation and subgrain formation play important roles in the mechanism of the acoustic softening effect, as demonstrated by Ngan et al. [20,21]. Generally, whether the metals will be permanently softened or hardened depends on the result of the ultrasonic effect on the microstructure, as pointed out by Lum et al. [18], residual hardening may occur in well-annealed metals with low dislocation density, while residual softening is more likely to occur in work-hardened metals or those subject to large deformation.

At present, investigations on the mechanisms behind the instantaneous or residual effects induced by UV primarily focus on uniaxial tensile or compression tests in which UV is applied longitudinally. In these tests, the direction of the ultrasonic load aligns with the tensile or compressive force, presenting advantages; for example, the sample is only subjected to the uniaxial stress state, and the experimental setup is relatively simple. However, in actual UV-assisted forming processes, most forming sheets experience a planar or three-dimensional stress state, including both in-plane and normal stresses. Technically, applying UV perpendicularly to the sample during the tensile test better simulates the stress states and plastic deformation behaviors encountered in actual forming processes, which is crucial for advancing UV-assisted forming techniques. In this paper, normal UV-assisted uniaxial tensile tests are carried out. The influences of ultrasonic amplitude and tensile strain rate on the plastic deformation behavior of pure titanium sheets are studied. Fracture morphologies are examined to reveal the influence of UV on fracture mode. EBSD and TEM microstructure analyses are conducted to investigate changes in crystallographic orientations, grain boundary angles, KAM distributions, GND density distributions, and IPF distributions, providing insights into the microstructure evolution of pure titanium sheets under normal UV.

## 2. Materials and Methods

### 2.1. Material Preparation

**Figure 1:**
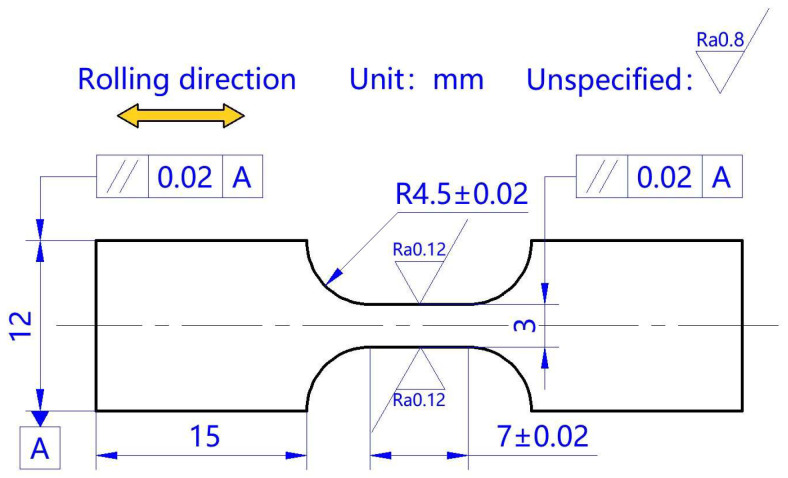
Dimensions of TA1 tensile sample.

Commercial pure titanium (Baoti Group Co., Ltd., Baoji, China) is widely used in aerospace and medical devices due to its excellent performance, such as high specific strength and desirable corrosion resistance. A cold-rolled TA1 titanium sheet with a thickness of 260 µm was used in this study. The alloy elements of the material are listed in Table 1. After annealing at 650 °C for 40 min in a vacuum environment (Hefei Kejing Material Technology Co., Ltd., Hefei, China), the cross-section of the TA1 sheet was polished and etched with a solution of 1 mL of HF (Chengdu Jinshan Chemical Reagent Co., Ltd., Chengdu, China), 2 mL of HNO_3_ (Chengdu Jinshan Chemical Reagent Co., Ltd., Chengdu, China), and 17 mL of deionized water(Hunan Zhongwo water environmental protection Technology Co., Ltd., Changsha, China) for 10–15 s to observe the microstructure using a metallographic microscope (Olympus (China) Co., Ltd., Shanghai, China). The grain size was manually measured using the intercept method according to ASTM E112 [22]. An average grain size of 19.4 μm was obtained by measuring three different areas in the sample. The tensile sample dimensions were proportionally scaled down according to similarity principles and the specifications of the national standard GB/T 228.1-2021 [23], and a gauge length and width of 7 mm and 3 mm were determined. Tensile samples were cut from the annealed TA1 sheet along the rolling direction using low-speed wire electrical discharging machining (Suzhou Baoma CNC equipment Co., Ltd., Suzhou, China), which is capable of reducing the surface roughness to Ra 0.12 μm. The dimensions and processing technical requirements are shown in Figure 1.

### 2.2. Experimental Setup

**Figure 2:**
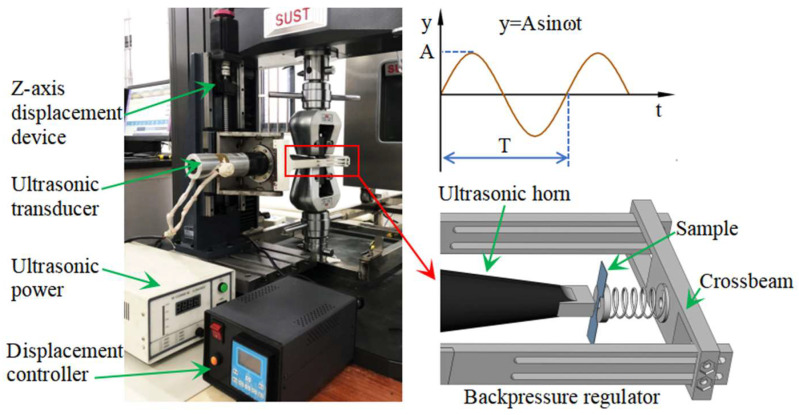
Normal UV-assisted tension system (A: amplitude; T: a period of vibration).

The normal UV-assisted tension system was developed on the basis of a universal testing machine (model: CMT5105G, load cell capacity: 100 kN, Zhuhai Sansi Taijie Electric equipment Co., Ltd., Zhuhai, China), as shown in Figure 2. The system comprises a Z-axis displacement device and controller (Shenzhen Youyi automation equipment Co., Ltd., Shenzhen, China), an ultrasonic transducer (frequency: 19.74 ± 0.2 kHz, amplitude: 0–15 µm, Shenzhen Rifa ultrasonic equipment Co., Ltd., Shenzhen, China), and an ultrasonic power supply (Shenzhen Rifa ultrasonic equipment Co., Ltd., Shenzhen, China). Unlike conventional longitudinal UV-assisted tension, the UV in this system is applied perpendicularly to the plane of the tensile sample. To prevent bending deformation from the ultrasonic horn, a backpressure device is implemented. This device counteracts the force from the ultrasonic horn by compressing a spring through the adjustment of the crossbeam position, thereby providing reverse support to the sample. To minimize the influence of backpressure on the mechanical properties during tensile tests, the pressure is set at approximately 5 N. Furthermore, to accommodate the upward movement of the gauge length area during deformation, the position of the ultrasonic transducer is adjusted upward at half the tensile speed of the sample. The displacement speed of the ultrasonic transducer is programmatically controlled by the Z-axis displacement device.

### 2.3. Experimental Scheme

To investigate the mechanical behavior and microstructure evolution of the TA1 pure titanium thin sheet under normal UV, uniaxial tensile tests were performed using varied ultrasonic amplitudes and strain rates. The specific parameters for UV and the loading methodology are presented in Table 2. SEM (Carl Zeiss Microscopy GmbH, Oberkochen, Germany) was utilized to examine the fracture morphologies of samples exposed to different ultrasonic amplitudes. Additionally, EBSD and TEM (FEI Tecnai F20, Thermo Fisher Scientific, Hillsboro, OR, USA) were employed to analyze the impact of normal UV on crystallographic orientation, grain boundary angles, KAM distribution, GND density distribution, and IPFs, all under consistent strain conditions (ε = 14%). It should be mentioned that all samples were preloaded to 5 N before tension to maintain uniform tensile conditions. Each tensile test was conducted at least 3 times to ensure reproducibility.

## 3. Results and Discussion

### 3.1. Acoustic Softening Effect

**Figure 3:**
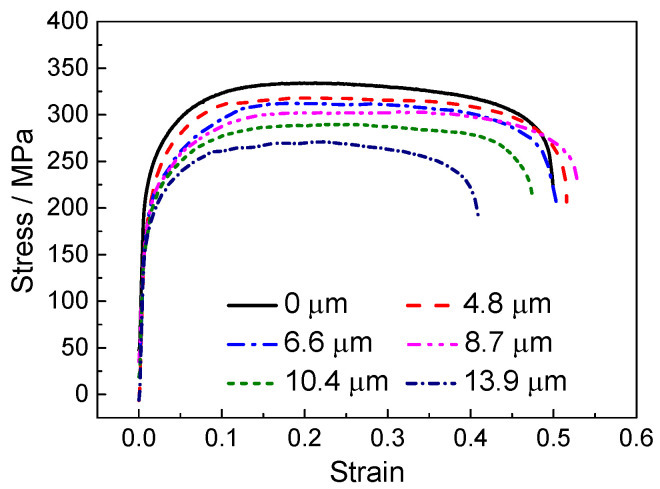
Influence of amplitude on flow stress.

**Figure 4:**
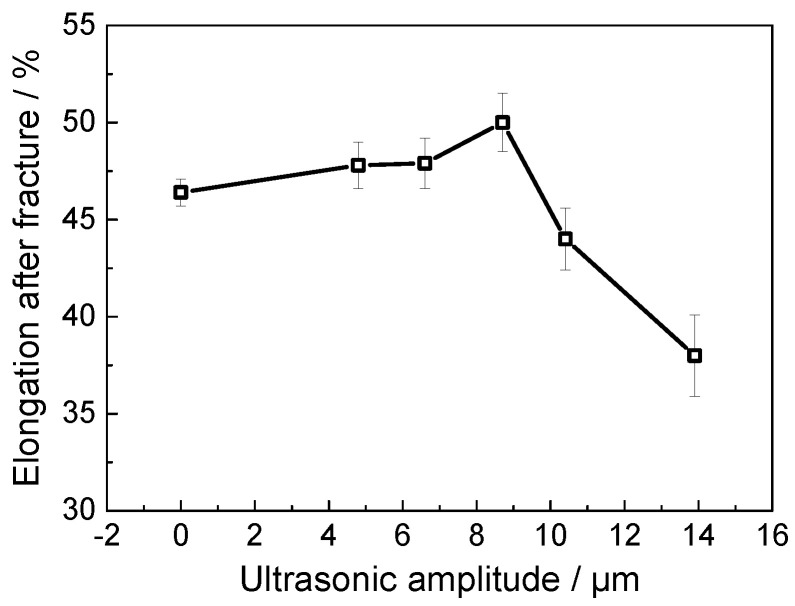
Elongations upon different amplitudes.

Figure 3 illustrates the stress–strain curves of tensile samples subjected to various ultrasonic amplitudes. A noticeable softening effect, characterized by a pronounced decrease in flow stress following UV application, is observed. Furthermore, the reduction in flow stress intensifies with increasing ultrasonic amplitude. At an amplitude of 13.9 µm, the stress reduction reaches approximately 20%. Wang et al. [13] also observed an enhancement in the acoustic softening effect with increased ultrasonic amplitude (ultrasonic energy density) in their research on pure titanium with longitudinal UV. Figure 4 displays the elongation after fracture under varying ultrasonic amplitudes. The elongation increases slightly with the amplitude, peaking at 50% when the amplitude is 8.7 µm. However, a further increase in amplitude results in a rapid decrease in elongation, falling to 38% when the amplitude is 13.9 µm. This pattern aligns with the findings of Meng et al. [25] for a superalloy thin sheet subjected to UV-assisted tension, in which the reduction in elongation at higher amplitudes was related to the transformation in fracture mode.

**Figure 5:**
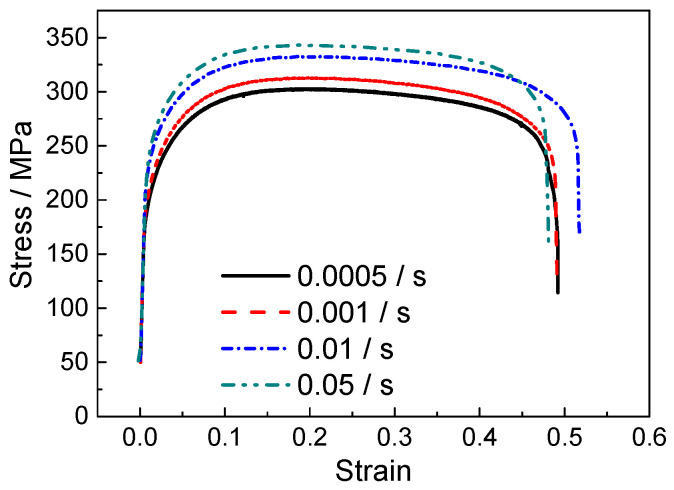
Flow stress without normal UV.

**Figure 6:**
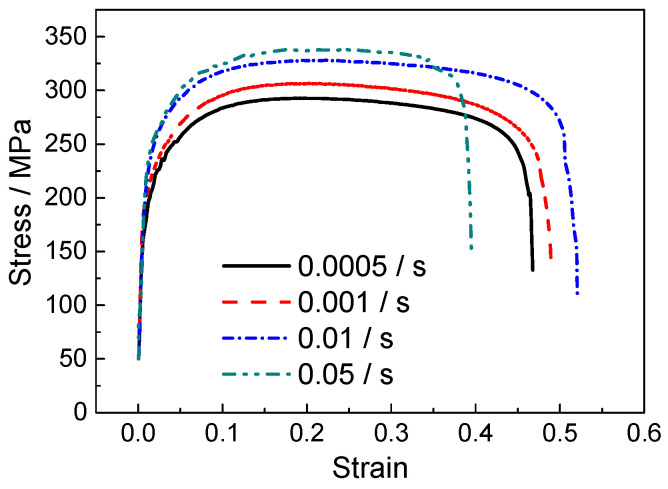
Flow stress with normal UV.

**Figure 7:**
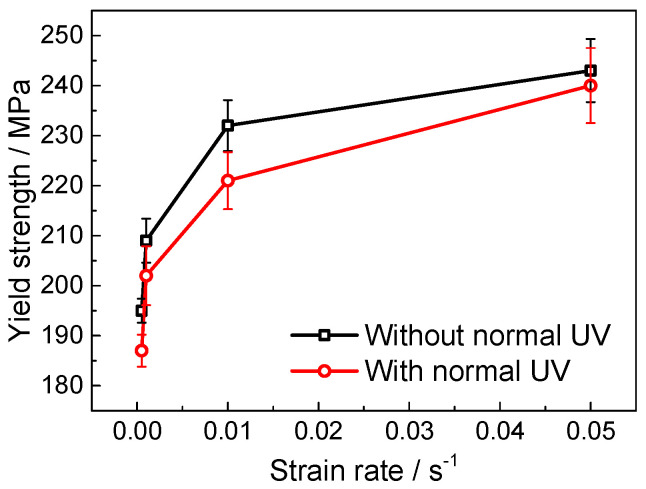
Yield strength under different strain rates.

Figure 5 and Figure 6 show the stress–strain curves under various strain rates in conditions with and without normal UV. It is evident that flow stress increases with the strain rate in both conditions. Similar to most crystalline metallic materials, titanium alloy undergoes severe plastic deformation at high strain rates, causing insufficient time available for dislocation slip and diffusion. This leads to significant dislocation pile-up and a rapid increase in local stress, resulting in stress inhomogeneity, which exacerbates material anisotropy or deformation incoherence, thus contributing to localized failure. At a strain rate of 0.05 s^−1^, the fracture strain notably decreases, especially under the condition with normal UV. Furthermore, the plastic deformation of polycrystalline materials is also affected by the coordination between intragranular and intergranular deformation. At higher strain rates, there is not enough time for this coordination, resulting in increased deformation resistance at the macroscopic level. Figure 7 presents the yield strength at different strain rates, indicating that normal UV significantly reduces yield strength across all tested strain rates, demonstrating a softening effect, especially at lower strain rates. At a strain rate of 0.05 s^−1^, the magnitude of softening is smaller. This reduction in softening capability at higher strain rates is primarily due to the limited absorption and conversion of ultrasonic energy by dislocations and other microstructure defects, thus diminishing the softening effect of UV at high strain rates.

### 3.2. Fracture Morphologies

**Figure 8:**
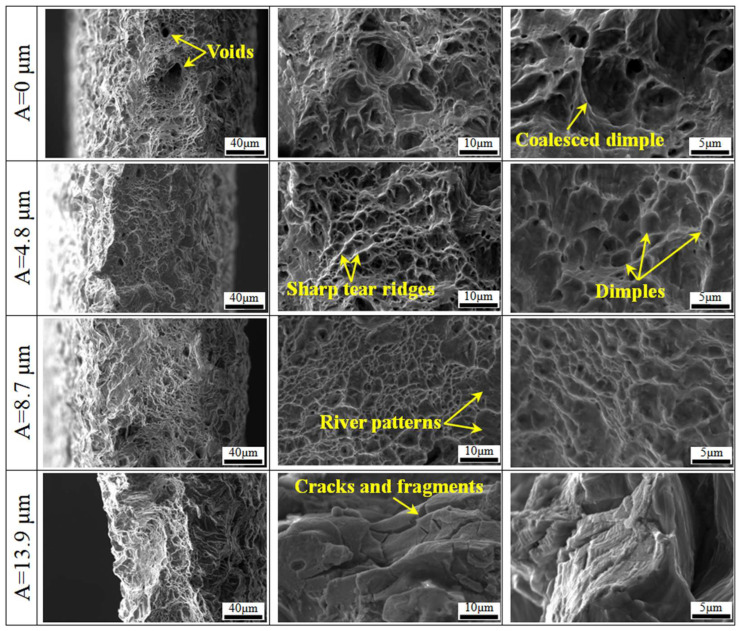
Fracture morphologies under various ultrasonic amplitudes.

Figure 8 presents the fracture morphologies of tensile samples subjected to various ultrasonic amplitudes. As can be seen, the sample strained without normal UV shows a fracture surface with straight slip bands interspersed with voids and dimples arranged irregularly. These voids vary significantly in depth and diameter, and the dimple density is low, showing multiple neighboring dimples coalescing, known as typical ductile fracture characterized by micro-void coalescence. At an ultrasonic amplitude of 4.8 µm, the diameter of voids decreases, and the number of equiaxed dimples increases, with high-magnification images revealing sharp tear ridges around the dimples. Increasing the amplitude to 8.7 µm results in a smoother fracture surface and an increased density of dimples, though with shallower depths. The tear ridges appear blunted, and isolated river patterns emerge in non-dimple areas, exhibiting quasi-cleavage fracture characteristics. At an amplitude of 13.9 µm, the fracture surface is uneven, the number of dimples decreases, and there is a prevalence of cracks and fragmented structures, indicating a brittle fracture characteristic.

Titanium alloys primarily undergo dislocation slip as the deformation mechanism during conventional tensile plastic deformation at room temperature. Owing to the limited slip systems, severe dislocation pile-up occurs, causing small voids to coalesce into larger ones, leading to fractures. When the normal UV with an appropriate amplitude is applied, dislocation pile-up can be alleviated, and the uniformity of plastic deformation is accordingly enhanced, as evidenced by the increased elongation shown in Figure 4. However, excessively high ultrasonic amplitudes lead to unsteady plastic deformation characterized by irregular plastic flow. Additionally, the increased strain rate at high amplitudes means some mobile dislocations do not have enough time to slip and become immobile, and this hinders the movement of other dislocations, leading to further dislocation pile-up and, subsequently, the nucleation and growth of micro-cracks, which manifest as brittle fracture characteristics at the macroscopic level. The above analysis indicates that applying an appropriate magnitude of normal UV can enhance the plastic deformation capability of a TA1 titanium sheet, which could be mutually verified by the result of elongation variations.

### 3.3. Microstructure Characterization

EBSD observations were carried out to reveal the microstructure evolution of the TA1 titanium sheet under normal UV-assisted tension. Samples under three conditions were considered: (1) unstrained, (2) 14% strain without normal UV, and (3) 14% strain with normal UV. The plane of observation was in the center of the deformed area perpendicular to the direction of the UV. The samples were manually polished with metallographic sandpaper with 500, 800, 1200, and 2000 grit in sequence until no scratch could be observed at 100 times magnification. Then, an automatic polishing machine (model: MoPao^®^2) was used to improve the surface finishing (rotary speed: 200 rpm, pressure: 8 N, time: 90 s) with 1 μm diamond suspension. An electropolishing method with 5% perchloric acid was further employed to achieve a mirror-like titanium sample. EBSD scans were carried out on a Zeiss Gmini 300 SEM equipped with an EBSD detector (Oxford symmetry 2) operating at an accelerating voltage of 20 kV, with a beam current of 14 nA and a working distance of 12 mm. The scanning position was about 50 μm deep below the surface. The sample was tilted at 70° to the horizontal axis for the EBSD scans, and a 0.5 μm step size and an 843 × 578 μm^2^ scanning area were used for all three samples. The EBSD data were post-processed using orientation imaging microscopy (OIM Analysis 6) data analysis software and ATEX software (version 4.11) [26].

**Figure 9:**
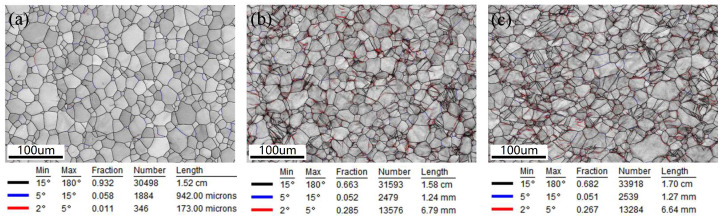
Grain boundary distributions: (**a**) unstrained sample, (**b**) 14% strained without normal UV, (**c**) 14% strained with normal UV.

**Figure 10:**
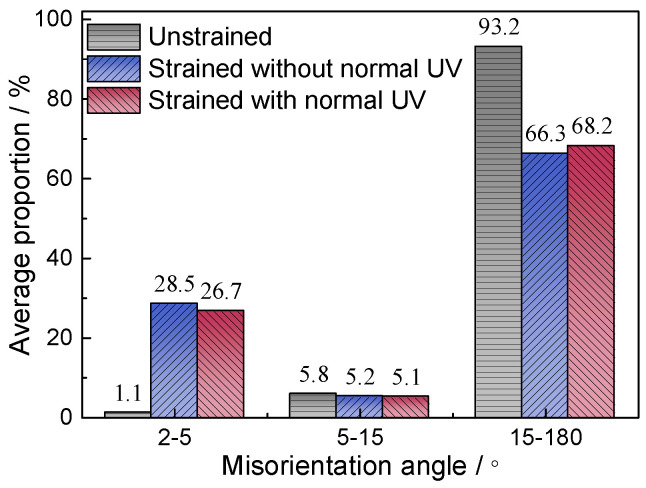
Average proportion of misorientation angles.

**Figure 11:**
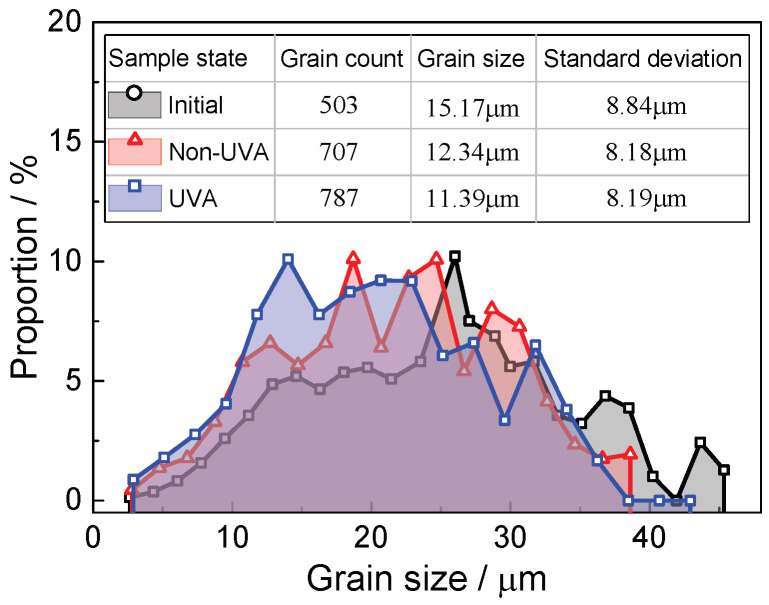
Grain statistics.

Figure 9 displays the statistical distribution of the grain boundary orientations for three samples, showing that the unstrained sample predominantly features high-angle grain boundaries (15°–180°), accounting for 93.2% of the total. After being strained to 14%, plastic deformation prompts significant dislocation movement, leading to dislocation pile-up and the formation of dislocation walls, which increase the number of low-angle grain boundaries (LAGBs) (2°–5°). The application of normal UV results in a decrease in the number of LAGBs compared to the sample strained without normal UV. As depicted in Figure 10, the proportion of LAGBs in the unstrained sample is 1.1%, which rises to 28.5% following strain without normal UV and then decreases to 26.7% after applying normal UV, similar to the observations reported by Wang et al. [13] and Dutta et al. [19]. This reduction is primarily attributed to the enhanced dislocation activity facilitated by UV energy, effectively reducing dislocation pile-up and promoting intragranular rotation. As a result, an increase in grain number and a decrease in average grain size are expected. As can be seen in Figure 11, the grain statistics show that the grain number and average grain size are 707 and 12.34 μm, respectively, while the values are 787 and 11.39 μm, respectively, when normal UV is applied, which demonstrates the above discussion.

**Figure 12:**
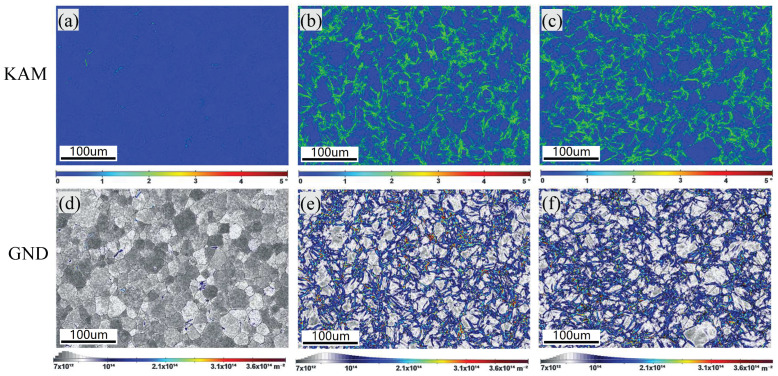
KAM and GND distributions: (**a**,**d**) unstrained sample, (**b**,**e**) 14% strained without normal UV, (**c**,**f**) 14% strained with normal UV.

**Figure 13:**
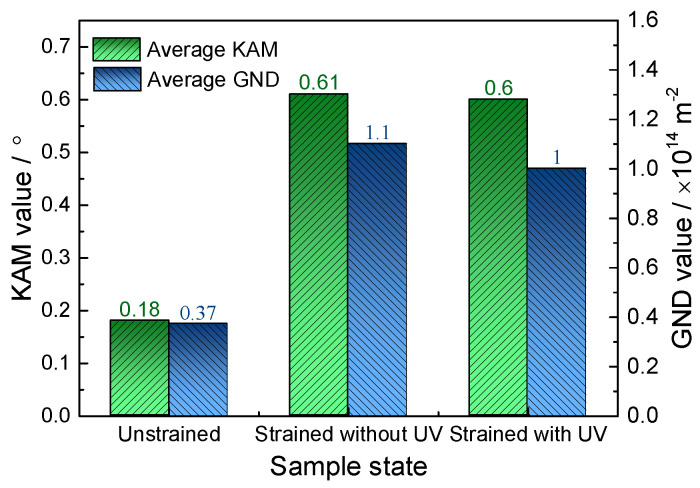
Average of KAM and GND density.

Figure 12 shows the EBSD maps of the unstrained sample (Figure 12a,d), the 14% strained sample without normal UV (Figure 12b,e), and the 14% strained sample with normal UV (Figure 12c,f). As expected, the unstrained sample shows almost no gradient in the KAM and GND distributions. By contrast, the 14% strained samples show remarkable orientation gradients in both the conditions with and without normal UV. The KAM and GND distribution maps also reveal considerable orientation gradients spreading along the grain boundaries. Figure 13 shows the average KAM and GND density values. For the unstrained sample, small KAM and GND density values indicate a small and randomly distributed local strain. After being strained to 14%, both values increase significantly, revealing a large plastic deformation. However, the KAM and GND density values decrease by 1.6% and 9.1%, respectively, when normal UV is superimposed. This could be explained by the preferential energy absorption by dislocations, which can increase the mobility of dislocations, thereby helping them overcome the lattice resistance. Another potential explanation for the decrease in the KAM and GND density values may be enhanced dipole annihilation caused by an oscillatory stress wave, as reported by Ngan et al. [27], who performed indentation experiments on aluminum excited by ultrasonic vibration. However, the dipole annihilation mechanism should be further verified, as there are different crystal structures with different slip systems between aluminum and titanium.

**Figure 14:**
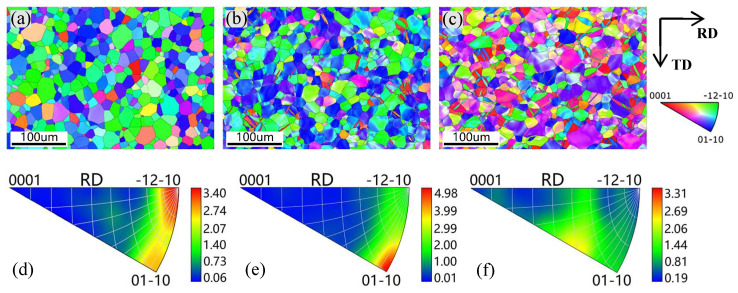
IPF-X distributions: (**a**,**d**) unstrained sample, (**b**,**e**) 14% strained without normal UV, (**c**,**f**) 14% strained with normal UV.

The IPF-X distributions of the samples under the three conditions are shown in Figure 14. The orientation distribution is visualized by color coding. It can be observed that there are significant changes in the microstructure and texture of the material under different conditions. The microstructure of the unstrained sample in Figure 14a shows only one color in each grain, indicating a uniform intragranular orientation distribution, which is the result of full annealing. It should be noted that the majority of the grains in Figure 14a appear green, representing a bias toward the {−12–10} texture component, as confirmed by the IPF in Figure 14d, where the strongest point is 3.40. For the strained samples, it is easy to notice that there is more than one color (orientation) within some of the grains in both cases, with and without normal UV, indicating that plastic deformation due to dislocation motion takes place within the grains. This may lead to the formation of a great number of LAGBs, as confirmed in Figure 10. For the 14% strained sample without normal UV in Figure 14b, it can be seen that the number of blue grains increases, revealing that the texture component changes from {−12–10} to {01–10}. Accordingly, the texture intensity increases to 4.98, as shown in Figure 14e. Thus, it can be inferred that the {01–10} slip system is activated as the dominant plastic deformation mechanism. However, the texture intensity decreases to 3.31 in the case with normal UV, and no evident preferred orientation is observed in Figure 14f. Also, a fully random orientation distribution is shown in Figure 14c. Since the direction of principal strain is along the rolling direction, it can be inferred that the {01–10} slip system is no longer dominant when normal UV is applied during tension. Specifically, plastic deformation coordination between grains is enhanced compared with the sample strained without normal UV, which could be attributed to the improvement in grain rotation induced by normal UV.

**Figure 15:**
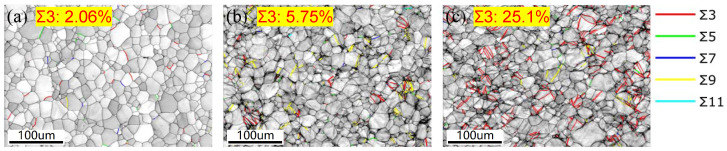
Twin boundary distributions: (**a**) unstrained sample, (**b**) 14% strained without normal UV, (**c**) 14% strained with normal UV.

**Figure 16:**
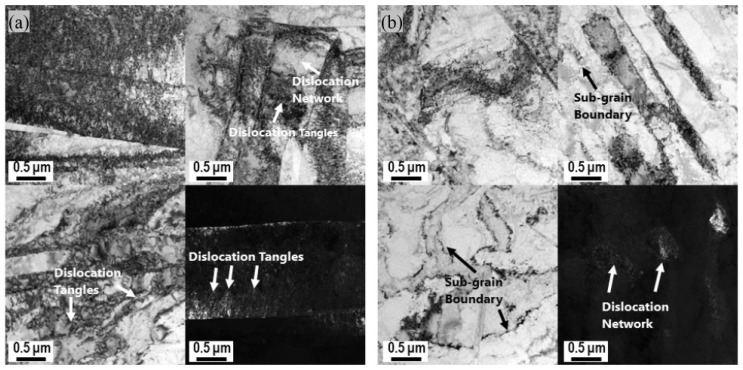
Bright-field and dark-field TEM images: (**a**) without normal UV, (**b**) with normal UV.

Figure 15 shows the twin boundary distributions of the three samples. The proportion of Σ3 twin boundaries is significantly increased from 5.75% to 25.1% due to normal UV. Generally, the twinning occurs under a high strain rate, and the critical resolved shear stress (CRSS) is much higher than the dislocation slip [28]. The deformation behavior under normal UV in this work is similar to that under a high strain rate. Therefore, twinning is enhanced during normal UV-assisted tension. Moreover, bright-field and dark-field TEM images of samples strained without and with normal UV are shown in Figure 16. The sample strained without normal UV exhibits a large number of dislocation pile-ups and tangles, both within the grains and at grain boundaries, with a relatively uniform distribution of dislocations. This clearly demonstrates the effective hindering effect of grain boundaries on dislocation motion. By contrast, the sample strained with normal UV has a lower internal dislocation density, and some dislocations form substructures such as dislocation walls and networks through pile-ups and tangles. Although certain amounts of dislocation pile-ups are also observed at grain boundaries, the accumulation degree is notably lower.

**Figure 17:**
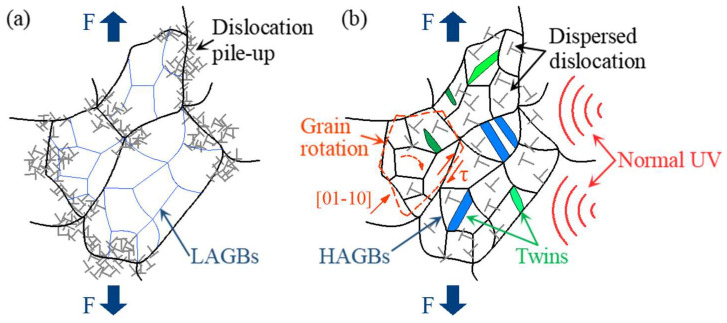
Schematic of deformation mechanism: (**a**) without normal UV, (**b**) with normal UV.

Comprehensively, the softening effect and residual effect observed in normal UV-assisted tension in this work are consistent with the results of most studies. However, the mechanism behind it is different. Generally, stress reduction induced by UV is considered a combination effect of elastic recovery-related stress superposition and dislocation motion-related acoustoplasticity. The stress superposition effect occurs solely due to longitudinal periodic ultrasonic loads that are aligned with the direction of the static tensile load. By contrast, normal ultrasonic load applied perpendicular to the static tensile load direction generally does not induce a stress superposition effect. Therefore, in experiments using normal UV, the acoustoplasticity effect becomes the primary cause of material softening, with other influences being minor. It is acknowledged that plastic deformation is caused by dislocation slips along various slip planes. Since few slip systems are present in HCP metals, titanium alloys are regarded as hard-to-deform materials. However, normal UV provides the energy required for dislocation movement and more deformation mechanisms, including the activation of grain rotation and twinning, which could make it easier to deform, as the schematic diagram shows in Figure 17. In contrast to conventional tension, applying normal UV changes the stress state of the material from a uniaxial stress state to a planar stress state, which more favorably promotes intergranular rotation and coordinated deformation. This transformation is likely to convert LAGBs into high-angle grain boundaries (HAGBs), as suggested by Liu et al. [16]. In addition, twinning is more likely to be activated at a high strain rate, as reported by Li et al. [29]. An increase in twin boundaries is observed under high strain rates induced by normal UV in this work. Also, the texture intensity decreases, and no evident preferred orientation is present under normal UV. Both the twinning and weakened grain orientation induced by normal UV are likely to increase the plastic deformation capability of titanium alloy. Das [30] also pointed out that ultrasonic vibration significantly enhanced the plastic deformation capability of Ti-6Al-4V alloy by facilitating dislocation motion, accelerating dynamic recovery processes and improving grain boundary sliding. All of the abovementioned mechanisms tend to result in a reduction in dislocation density, as shown in the KAM and GND results in Figure 13, and, consequently, a decrease in pile-ups along grain boundaries.

## 4. Conclusions

The effect of normal UV on the mechanical properties and microstructure evolution of a TA1 titanium sheet was systematically investigated. Compared with traditional UV-assisted tension, the normal UV-assisted tension method provides a superior simulation of deformation behavior in practical forming with enhanced vibration stability, albeit with a relatively complex experimental setup. From the experimental results, the following conclusions can be drawn:

1. Based on normal UV-assisted uniaxial tensile testing, the acoustic softening effect increases with increasing the ultrasonic amplitude from 4.8 μm to 13.9 μm, while the elongation shows a maximum value of 50% at an ultrasonic amplitude of 8.7 μm.
2. Combined with the elongation results, fracture morphologies obtained from SEM show that the fracture mode of the TA1 changes from ductile fracture to brittle fracture with increasing ultrasonic amplitude.
3. Instead of stress superposition, acoustoplasticity is considered the only deformation mechanism for the TA1 titanium sheet under normal UV.
4. EBSD and TEM observations show that superimposed normal UV can lead to grain refinement and a decrease in LAGB proportion and KAM and GND density, resulting in homogeneous plastic deformation and flow stress reduction.
5. The IPFs reveal that the texture intensity decreases from 4.98 to 3.31 when normal UV is superimposed. Concurrently, the proportion of Σ3 twin boundaries significantly increases from 5.75% to 25.1%, suggesting enhanced activation of multiple slip systems and additional plastic deformation mechanisms, such as twinning and grain rotation, contributing to improved deformation uniformity.

## Figures and Tables

**Table 1:** Alloy elements of TA1 alloy sheet (wt.%) [24].

Ti	Fe	C	N	H	O
≥99.50	≤0.25	≤0.10	≤0.03	≤0.015	≤0.20

**Table 2:** Experimental condition and parameters.

Group	Amplitude (μm)	Duration (s)	Tensile Speed (mm·min^−1^)	Strain Rate (s^−1^)
1 (Amplitude)	4.8	Throughout	5	0.012
	6.6			
	8.7			
	10.4			
	13.9			
2 (Strain rate)	4.8	Throughout/ None	0.21	0.0005
			0.42	0.001
			4.2	0.01
			21	0.05

## Data Availability

The original contributions presented in the study are included in the article. Further inquiries can be directed to the corresponding author.
